# Health-Related Indicators Measured Using Earable Devices: Systematic Review

**DOI:** 10.2196/36696

**Published:** 2022-11-15

**Authors:** Jin-Young Choi, Seonghee Jeon, Hana Kim, Jaeyoung Ha, Gyeong-suk Jeon, Jeong Lee, Sung-il Cho

**Affiliations:** 1 Department of Public Health Science Graduate School of Public Health, Seoul National University Seoul Republic of Korea; 2 Department of Nursing College of Natural Science, Mokpo National University Mokpo Republic of Korea; 3 Department of Nursing College of Health and Medical Science, Chodang University Muan Republic of Korea; 4 Institute of Health and Environment Seoul National University Seoul Republic of Korea

**Keywords:** digital public health, earable, wearable, biomarker, health status, disease monitoring, prevention strategy, Internet of Things, systematic review, mobile phone

## Abstract

**Background:**

Earable devices are novel, wearable Internet of Things devices that are user-friendly and have potential applications in mobile health care. The position of the ear is advantageous for assessing vital status and detecting diseases through reliable and comfortable sensing devices.

**Objective:**

Our study aimed to review the utility of health-related indicators derived from earable devices and propose an improved definition of disease prevention. We also proposed future directions for research on the health care applications of earable devices.

**Methods:**

A systematic review was conducted of the PubMed, Embase, and Web of Science databases. Keywords were used to identify studies on earable devices published between 2015 and 2020. The earable devices were described in terms of target health outcomes, biomarkers, sensor types and positions, and their utility for disease prevention.

**Results:**

A total of 51 articles met the inclusion criteria and were reviewed, and the frequency of 5 health-related characteristics of earable devices was described. The most frequent target health outcomes were diet-related outcomes (9/51, 18%), brain status (7/51, 14%), and cardiovascular disease (CVD) and central nervous system disease (5/51, 10% each). The most frequent biomarkers were electroencephalography (11/51, 22%), body movements (6/51, 12%), and body temperature (5/51, 10%). As for sensor types and sensor positions, electrical sensors (19/51, 37%) and the ear canal (26/51, 51%) were the most common, respectively. Moreover, the most frequent prevention stages were secondary prevention (35/51, 69%), primary prevention (12/51, 24%), and tertiary prevention (4/51, 8%). Combinations of ≥2 target health outcomes were the most frequent in secondary prevention (8/35, 23%) followed by brain status and CVD (5/35, 14% each) and by central nervous system disease and head injury (4/35, 11% each).

**Conclusions:**

Earable devices can provide biomarkers for various health outcomes. Brain status, healthy diet status, and CVDs were the most frequently targeted outcomes among the studies. Earable devices were mostly used for secondary prevention via monitoring of health or disease status. The potential utility of earable devices for primary and tertiary prevention needs to be investigated further. Earable devices connected to smartphones or tablets through cloud servers will guarantee user access to personal health information and facilitate comfortable wearing.

## Introduction

### Background

ITs for monitoring health-related indicators have been developed and are continually being upgraded. Wearable devices are a major part of new health monitoring schemes related to out-of-hospital health care, occupational medicine, and sports science and technology [1-3]. As the world’s population continues to age, the benefits of the improved cost-effective health care that this technology can provide will increase [4,5]. The biomarkers that can be detected by diverse wearable biosensors include electrocardiography (ECG) data, heart rate (HR), blood pressure, body and skin temperature, and respiration rate [1,6].

The Internet of Things (IoT) has been combined with various technologies used in our daily lives. The IoT is a versatile platform that can obtain data from an object and transmit them to an internet server to manage status in real time [7,8]. Wearable IoT is a branch of IoT technology applied to networking and communication of wearable devices [9]. Wearable IoT devices can track physiological activity in an interconnected manner. Wearability should be considered when designing monitoring systems; it is important that the devices are small [9,10].

The ear is a promising location for biosensors detecting critical conditions or diseases given its potential for noninvasiveness [3,11]. Ear electroencephalography (EEG) is used to calculate the surface potential of the ear, which varies with ear topography [12]. The tympanic membrane is associated with the vasculature of the ear canal, to which sensors can be attached to detect physiological signals. When blood is discharged from the basilar artery to the tympanic membrane through the internal carotid artery, the flow through the arteries involves anastomoses made by several branches of the external carotid, anterior tympanic, posterior auricular, stylomastoid, and maxillary arteries [3,13,14]. The internal carotid artery passes through the circle of Willis and brain, and *bifurcation anastomosis* plays an important role in monitoring physiological biomarkers related to the blood supply to the hypothalamus for thermoregulation. Measurements of core temperature in 2 parts of the ear canal supplying blood to the brain confirmed thermal equilibrium. Blood flow is a reliable indicator of ear vascularization even when users are sick [3,15,16].

Wearable devices that are worn around the ear are named “earable” or “hearable.” Kurosawa et al [17] first proposed the term “earable” in 2017 to refer to a novel earphone-type wearable sensor. On the basis of this prototype, they developed several earable models for various uses [18-21]. Ota et al [22] expanded the earable device concept, defining such a device as a “wearable electronic designed to be worn around the ear.” Hunn [23] coined the term “hearable” in 2014 to refer to a device “that fits in or on an ear that contains a wireless link, whether that’s for audio, or remote control of audio augmentation.” The potential use of hearable devices for the measurement of vital signs has been reviewed extensively [3,24]. Hearables are a promising type of hearing device for individuals with hearing loss [25]. As “earable” indicates only the device position whereas “hearable” suggests both ear position and involvement in hearing function, we conceptualize hearables as a subset of earables, which in turn are a subtype of wearables (Multimedia Appendix 1). According to this view, the recently increasing interest in new devices worn in or around the ear is related mainly to earables as their relevant functions are not limited to hearing.

In total, 2 recent reviews have demonstrated that valuable information can be obtained using earables, although the term “hearables” was used in these reviews. In 2020, Mase et al [3] reviewed the use and performance of hearable-based physiological monitoring. Among the 39 articles that they identified, the main physiological parameters described were temperature (24 articles), HR or pulse rate (12 articles), and oxygen saturation (3 articles) monitored in daily life. In 2021, Ne et al [24] evaluated the challenges and capabilities of physiological signal monitoring. They reported that ear signal acquisition yielded satisfactory outcomes relative to gold-standard monitoring. For better application in the future, improvements in wireless connectivity, battery life, the impacts of motion and environmental artifacts, and comfort are required.

An important function of wearable devices is health status monitoring, which enables preventive action. For example, several wrist- and arm-worn devices have been developed to detect epileptic seizures. The signals from the device sensor are based on electrodermal activity and HR changes; these are used to detect the preictal state before a seizure. Seizure events can be detected based on shaky motor movements and then logged and reported [26]. A second example concerns dietary management. Studies are being performed to develop a technology to detect food intake patterns through a wearable device. Examples include an in-ear microphone that detects and characterizes food intake, a watch-type device that tracks wrist movements during meals, and a necklace-like wearable sensor system for automatic ingestion monitoring. These technologies can be applied in dietary interventions and as tools to improve dietary behavior, which consequently contributes to the prevention or reduction of the incidence of obesity and eating disorders [27]. Risk factors for noncommunicable diseases (eg, physiological factors, tobacco use, alcohol abuse, unhealthy diet, lack of physical activity, and overweight or obesity) can be controlled through device reminders promoting healthy behaviors [4]. Although earable devices can measure diverse biomarkers, their application in disease prevention has not been well studied.

To facilitate the preventive uses of earable devices, broad preventive strategies are required. The significance of prevention using earable devices follows the vision of digital public health (DPH), which aims to expand health promotion from the individual level to the population level using information and communications technology. DPH involves disease prevention, the facilitation of population participation, the promotion of value-based health care, and the provision of universal health coverage [28]. The accessibility and functionality of earable devices are promising features for prevention in DPH.

The Centers for Disease Control and Prevention defines the prevention stages outlined in Textbox 1 [29,30].

The DPH vision suggests that earable devices may be used as accessible instruments that expand the scope of preventive services and activities. The use of earable devices for the continuous monitoring of diverse essential biomarkers may provide additional possibilities at each stage of prevention. Advances in earable devices may facilitate disease prevention by promoting healthier lifestyles. Thus, a review of the existing research on the applications of earable devices is needed.

**Definitions of prevention stages according to the Centers for Disease Control and Prevention.**

**Definitions of prevention**

• Primary prevention: intervening before the disease process begins through measures such as vaccination, adjusting dietary habits, or quitting tobacco use

• Secondary prevention: screening for early diagnosis of diseases (eg, mammography for breast cancer and regular blood pressure testing for cardiovascular disease)

• Tertiary prevention: slowing down or attenuating disease progression via different measures after the onset of the disease (eg, chemotherapy for cancer, rehabilitation for injuries, and screening for complications)

### Objectives

In this study, we first conducted a systematic review of the application of earable devices for prevention. Second, we proposed updated definitions of the prevention stages in which earable devices may be applied. Third, we explored future research directions for wearable devices to maximize IoT-related functionality in the health care field. As we focused on applications related to prevention, we excluded studies that were related only to hearing problems.

## Methods

### Design

The PRISMA (Preferred Reporting Items for Systematic Reviews and Meta-Analyses) format [31] was used to summarize the major studies related to the use of earable devices for health care. This review was conducted to identify the health-related indicators measured using earable devices and the current state of these devices’ development and use.

### Search Strategy

We conducted a comprehensive search of the PubMed, Web of Science, and Embase databases to identify relevant articles published in English between 2015 and 2020 (Table S1 in Multimedia Appendix 2). We searched publication titles, keywords, and abstracts. We used standardized Medical Subject Heading keywords provided by PubMed and Emtree in Embase as well as free-text words.

We used the terms “earable” OR “hearable” OR (“ear” AND [“wearable” OR “wearability”]) in all databases. As alternative terms for “wearable,” related Medical Subject Heading terms (“wearable electronic devices”) and Emtree terms (“wearable computer” and “wearable sensor”) were also used.

### Inclusion and Exclusion Criteria

In total, 1193 articles were identified. After excluding duplicate publications, 83.4% (995/1193) of the articles were screened. Articles with abstracts not aligning with the study objectives or inclusion criteria were also omitted, and 51 articles were ultimately included in the analysis.

The review included original articles that focused on the physiological and physical influences of the body (except for auditory function) in human participants that were published in full-text format in English. We excluded articles not related to humans (eg, animal studies) and those that focused solely on ear function or ear disease (eg, hearing loss, hearing aids, and hearing aid signal processing). Studies related to hearing were excluded as such functions were already reviewed in a recent study [25]. In addition, editorials and letters were excluded (Table S2 in Multimedia Appendix 2).

### Study Selection

Three authors (JC, SJ, and HK) selected articles for inclusion in four steps. First, they independently identified and screened publication titles, keywords, and abstracts to identify relevant articles. Second, the abstracts of relevant articles were screened for eligibility. Third, the full-text versions of eligible articles were selected according to the inclusion and exclusion criteria by consensus. Finally, additional articles were identified by manual searching of the reference lists of the relevant articles under consideration.

### Data Collection and Extraction

The following five main categories of data were extracted from the selected studies (Multimedia Appendix 3 [12,18-22,32-76]): (1) publication details (first author and publication year), (2) target health outcomes (study outcomes related to health status or targeted disease), (3) biomarkers (biosignals produced from sensors to detect the target health outcomes), (4) sensor type (energy form used for data collection during health status analyses), (5) sensor position (earable sensor position used for data collection), and (6) prevention stage (most relevant prevention stage for the earable device).

Three authors (JC, SJ, and HK) extracted the data using a common data collection form. To validate the extraction, a test set of 20% (10/51) of the selected articles was compiled using systematic random sampling. The 3 authors independently extracted data from the test set and had a consensus discussion to standardize the extraction methods. Data extraction was finalized according to the resulting standard procedure.

### Quality Assessment

The quality of this systematic review was assessed using AMSTAR-2 (A Measurement Tool to Assess Systematic Reviews) [77]. The quality of the review satisfied 9 out of 13 items that applied to systematic reviews without meta-analysis (Table S3 in Multimedia Appendix 2).

## Results

### Article Characteristics

**Figure 1:**
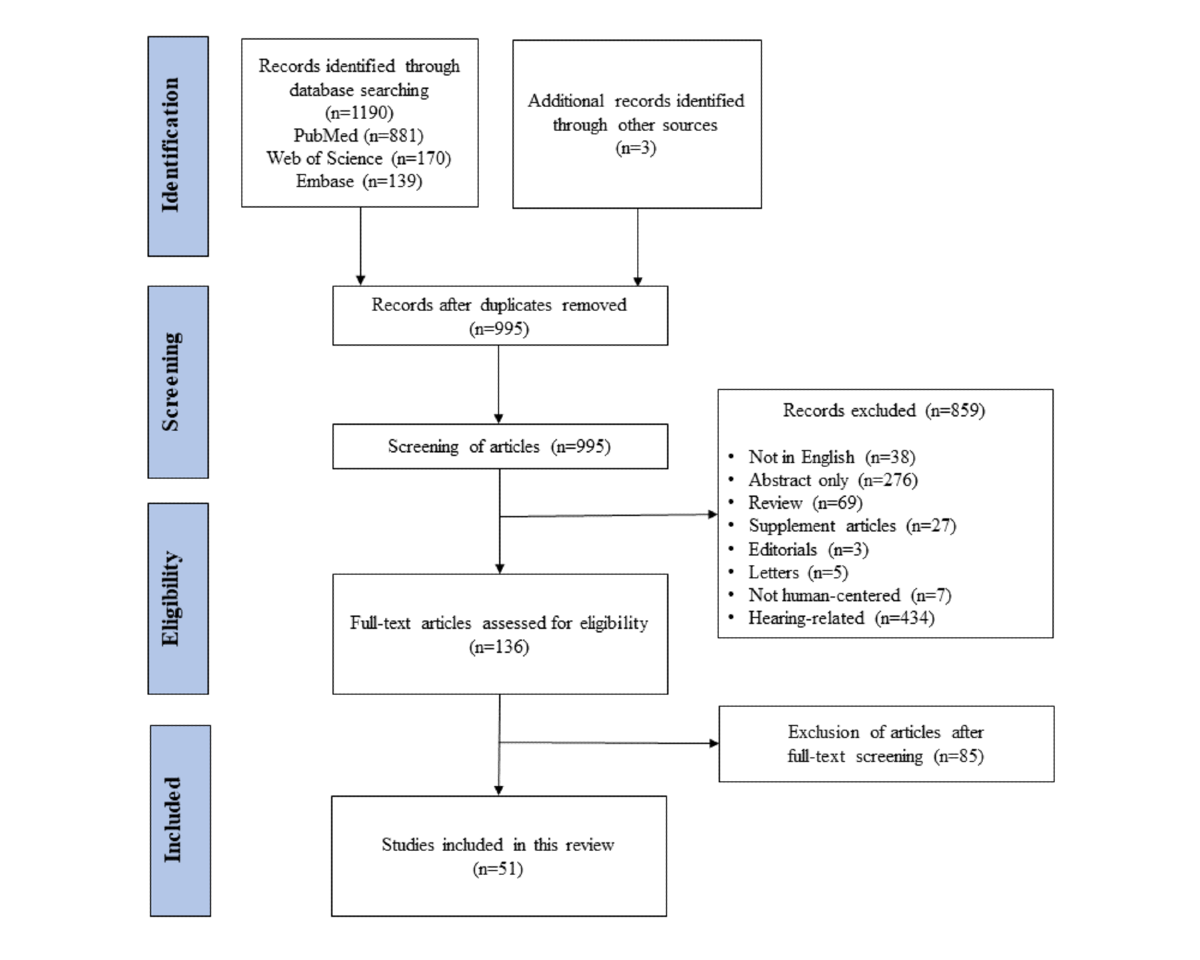
Flowchart of the search strategy and literature selection process.

The literature review identified 1193 articles as potentially relevant and finally included 51 articles that fulfilled the inclusion criteria (Figure 1).

The number of published articles was relatively low in 2015 and 2016 but sharply increased since 2017 to >10 per year. The research topics were diversified and were particularly related to health-related indicators and sensor types, whereas the position of the earable device largely remained constant—in the ear canal—in most studies (26/51, 51%; Multimedia Appendix 4).

We observed that research on earable devices for health promotion, health monitoring and diagnosis, treatment, and rehabilitation is progressing. Table 1 classifies the earable devices according to five domains: “target health outcomes,” “biomarkers,” “sensors,” “sensor position,” and “preventive stage.”

Reported uses of earable devices were diet-related activity monitoring (9/51, 18% of the articles) [19,32-39]; brain status monitoring (7/51, 14% of the articles) [40-45,78]; cardiovascular disease (CVD) monitoring (5/51, 10% of the articles) [46-50]; central nervous system (CNS) disease monitoring and diagnosis (5/51, 10% articles) [51-54,79]; head injury monitoring (4/51, 8% of the articles) [55-58]; and monitoring of heart status [59-61], respiration [20,62], and sleep disorders [63,64] (7/51, 14% of the articles). Earable devices were used to monitor multiple diseases and health conditions in 16% (8/51) of the studies, namely, brain, cardiac, and respiratory functions [65]; cardiovascular status, sweating, and motion [66]; HR and breathing rate [67]; respiration and posture [21]; metabolic functions in relation to fever, insomnia, fatigue, and depression [22]; gait classification [68]; cardiovascular, metabolic, and mental disorders, including stress and pain response [69]; and chronic stress, cognitive dysfunctions, depression, and CVD [70]. In another 12% (6/51) of the studies, earable devices were used to monitor various aspects of health status, namely, thermoregulation [71], fertility [72], heat stress [73], tongue movements [18], facial expressions [74], and physical activity [75].

EEG was used for monitoring in 22% (11/51) of the studies [40,41,43-45,51-54,63,78], body movements were monitored in 12% (6/51) of the studies [18-21,33,75], and body temperature was monitored in 10% (5/51) of the studies [22,64,71-73]. Photoplethysmography (PPG) was used for monitoring in 8% (4/51) of the studies [32,48,60,69], acceleration stress was monitored in 8% (4/51) of the studies [55-58], and ECG was used for monitoring in 6% (3/51) of the studies [49,50,59]. A total of 25% (13/51) of the articles reported the monitoring of multiple exposure- and disease-related biomarkers (ie, EEG outputs, breathing signals, and mechanical plethysmography [MPG] outputs [65]; ECG outputs, lactate levels, and head acceleration [66]; ECG, ballistocardiography, and PPG outputs [46]; PPG and bioacoustics outputs and vibrations [36]; PPG and bioacoustics outputs [34]; ear canal shape, electromyography [EMG] outputs, and occlusal force [39]; acceleration stress, body temperature, and HR variability [HRV] [68]; EEG, electro-oculography, and EMG outputs [42]; EEG and ECG outputs [70]; ear canal shape, muscle movement, and acoustic signals [35]; EMG outputs, ear canal pressure, and muscle movement [38]; PPG outputs and air pressure [61]; and body potential, EMG outputs, and capacitance [74]). Other biomarkers measured in the studies included oxygen saturation [62], caloric vestibular stimulation [79], breathing signals [67], ear pulse waves (EPWs) [47], and EMG outputs [37].

Electrical sensors were described in 37% (19/51) of the articles on device development [37,40-45,47,49-54,59,63,70,74,78]. Photosensors were described in 18% (9/51) of the articles [18-20,32,33,48,60,62,69], mechanical sensors were described in 10% (5/51) of the articles [45,55-58], and thermal sensors were described in 6% (3/51) of the articles [72,73,79]. Acoustic sensors were used to monitor cardiac and respiratory status to improve patient safety [67]. The aforementioned sensors were attached to the devices in isolation. A total of 27% (14/51) of the articles described the use of more complex sensors, including 12% (6/51) of cases in which electrical, mechanical, optical, and pressure sensors were used in combination [38,39,46,61,65,68] and 6% (3/51) of cases in which sensors of electricity and heat were used [22,64,71]. Amperometric and potentiometric sensors were used in 1 multisensory device [66]. Another sensor combination was optical plus acoustic or mechanical sensors [21,34-36]. In total, 51% (26/51) of the articles described devices for the ear canal, which was the primary device location, used in almost half of the studies [18-22,33,38,40,42-45,47,50,58,60-65,67,68,71,72,74]. The devices were positioned behind the ear in 16% (8/51) of the studies [35,37,51,54-57,59] and around the ear [41,66,70] or on the earlobe [32,48,69] in 12% (6/51) of the studies. The devices were placed in the inner ear in 4% (2/51) of the studies [73,75] and in the concha in 2% (1/51) of the studies [34]. In total, 16% (8/51) of the articles described multiple body positions for sensor attachment. In 8% (4/51) of the studies, sensors were attached to the ear canal and concha [52,53,78,79]. Additional locations for sensor attachment included around the ear [36], near the ear adjacent to the mastoid and neck [46], in the oral cavity at the masseter muscle [39], and on the head [49].

Most of the studies (35/51, 69%) were concerned with secondary prevention [21,22,39-41,44-71,73,78], although 24% (12/51) were related to primary prevention [20,32-38,42,43,72,75], and 8% (4/51) were related to tertiary prevention [18,19,74,76].

**Table 1:** Characteristics of the earable devices described in the articles (N=51).

Health-related characteristics			Studies, n (%)
**Target health outcomes**			
	Diet-related	9 (18)	
	Brain status	7 (14)	
	Cardiovascular disease	5 (10)	
	Central nervous system disease	5 (10)	
	Head injury	4 (8)	
	Heart status	3 (6)	
	Respiration	2 (4)	
	Sleep disorder	2 (4)	
	Combination	8 (16)	
	Other	6 (12)	
**Biomarker**			
	EEG^a^	11 (22)	
	Body movements	6 (12)	
	Body temperature	5 (10)	
	PPG^b^	4 (8)	
	Acceleration stress	4 (8)	
	ECG^c^	3 (6)	
	Combination	13 (25)	
	Other	5 (10)	
**Sensor type**			
	Electrical	19 (37)	
	Photo	9 (18)	
	Mechanical	5 (10)	
	Thermal	3 (6)	
	Acoustic	1 (2)	
	Combination	14 (27)	
**Sensor position**			
	Ear canal	26 (51)	
	Behind the ear	8 (16)	
	Around the ear	3 (6)	
	Earlobe	3 (6)	
	Inner ear	2 (4)	
	Ear concha	1 (2)	
	Multiple	8 (16)	
**Prevention stage**			
	Primary	12 (24)	
	Secondary	35 (69)	
	Tertiary	4 (8)	

^a^EEG: electroencephalography.

^b^PPG: photoplethysmography.

^c^ECG: electrocardiography.

### Target Health Outcomes

Tables 2-4 show the cross-tabulated data for target health outcomes and biomarkers. In total, 22% (11/51) of the studies measured health outcomes related to the brain and head using different sensor types (electric and mechanical) and biomarkers (EEG and body movements). A total of 14% (7/51) of the studies used electric sensors for detecting EEG signals [40-45,78], and 8% (4/51) used mechanical sensors capturing acceleration stress [55-58].

Three different sensor types (electric, photo, and a combined sensor [46]) and four different biomarkers (ECG, PPG, EPWs, and combined biomarkers) were used to measure CVD-related outcomes in 10% (5/51) of the studies. In total, 4% (2/51) of the articles reported the use of an electric sensor for ECG detection [49,50], 2% (1/51) of the articles described the use of a photosensor for PPG detection [48], and another article (1/51, 2%) described the use of an electrical sensor for EPW detection [47].

Two sensor types (electrical and thermal) and two biomarkers (EEG and caloric vestibular stimulation) were used in 10% (5/51) of the studies to measure the outcomes of CNS disease. A total of 8% (4/51) of the studies investigated the utility of electrical sensors for detecting EEG signals in the CNS [51-54], and 2% (1/51) of the studies used thermal sensors for caloric vestibular stimulation as a disease treatment [79].

In total, 10% (5/51) of the studies explored dietary outcomes using combinations of photosensors, acoustic sensors, and mechanical sensors [34-36,38,39]. Four biomarkers (body movements, PPG and EMG outputs, and a combined biomarker) were used for PPG, bioacoustic signaling, pressure, strain, and vibration detection. In addition, photosensors were used to detect body movement in 4% (2/51) of the studies [19,33], and PPG [32] and EMG [37] outputs were used in 2% (1/51) of the studies each.

A total of 14% (7/51) of the studies (aiming to monitor heart status, CVD, metabolic diseases, and mental disorders) involved the use of electric sensors and photosensors for ECG and PPG detection [32,48-50,59,60,69]. A photosensor was included in a device to detect respiration indicators, and body movements and oxygen saturation were used as biomarkers [62]. Earable devices have also been developed to monitor sleep status, including electric and multisensory devices (eg, “auditory temperature”) [64]. Biomarkers were used by some devices to detect sleep disorders, EEG, and body temperature. Various sensors targeting different biomarkers have been used to detect health status indexes. For example, ECG was used to measure cardiovascular activity, sweating, and motion [66]. Combinations of electric, amperometric, potentiometric, and mechanical sensors were used by some devices for measuring various target health outcomes and vital functions (eg, brain, cardiac, and respiratory functions) via biomarkers such as EEG, MPG, and bioacoustic (breathing) signals.

Health conditions monitored using the devices included metabolic disorder, fever, fatigue, insomnia, and depression [22,69]. Core body temperature was used as a biomarker for a device with a photosensor and mechanical sensor [22,71,73]. Some studies were concerned with novel target health outcomes, including respiration and posture status measured based on body movements determined by a combination of a photosensor and mechanical sensor [20,62]; heart and breathing rates measured using bioacoustic signals and acoustic sensors [67]; temporomandibular joint function determined based on the number of chews and changes in the shape of the ear canal; and EMG signals and occlusal force measured using a combination of photo, electric, and pressure sensors [19]. We also identified studies that measured tongue movements and changes in the shape of the ear canal as detected by a photosensor with the goal of overcoming physical disabilities [18]. A total of 6% (3/51) of the studies monitored fertility, heat stress, and thermoregulation based on body temperature using a single thermal sensor or a combination of electric and thermal sensors [71-73].

**Table 2:** Target health outcomes (n≥5) by sensor type and biomarkers.

		Target health outcomes, n (%)			
		Diet-related (n=9)	Brain status (n=7)	Cardiovascular disease (n=5)	Central nervous system disease (n=5)
**Sensor types**					
	Electrical	1 (11)	7 (100)	3 (60)	4 (80)
	Photo	3 (33)	—^a^	1 (20)	—
	Mechanical	—	—	—	—
	Thermal	—	—	—	1 (20)
	Acoustic	—	—	—	—
	Combination	5 (56)	—	1 (20)	—
**Biomarkers**					
	EEG^b^	—	6 (86)	—	4 (80)
	Body movements	2 (22)	—	—	—
	Body temperature	—	—	—	—
	ECG^c^	—	—	2 (40)	—
	Acceleration stress	—	—	—	—
	PPG^d^	1 (11)	—	1 (20)	—
	Combination	5 (56)	1 (14)	1 (20)	—
	Other	1 (11)	—	1 (20)	1 (20)
Total (column; N=51)		9 (18)	7 (14)	5 (10)	5 (10)

^a^Not available.

^b^EEG: electroencephalography.

^c^ECG: electrocardiography.

^d^PPG: photoplethysmography.

**Table 3:** Target health outcomes (n<5) by sensor type and biomarkers.

		Target health outcomes, n (%)			
		Head injury (n=4)	Heart status (n=3)	Respiration (n=2)	Sleep disorder (n=2)
**Sensor types**					
	Electrical	—^a^	1 (33)	—	1 (50)
	Photo	—	1 (33)	2 (100)	—
	Mechanical	4 (100)	—	—	—
	Thermal	—	—	—	—
	Acoustic	—	—	—	—
	Combination	—	1 (33)	—	1 (50)
**Biomarkers**					
	EEG^b^	—	—	—	1 (50)
	Body movements	—	—	1 (50)	—
	Body temperature	—	—	—	1 (50)
	ECG^c^	—	1 (33)	—	—
	Acceleration stress	4 (100)	—	—	—
	PPG^d^	—	1 (33)	—	—
	Combination	—	1 (33)	—	—
	Other	—	—	1 (50)	—
Total (column; N=51)		4 (8)	3 (6)	2 (4)	2 (4)

^a^Not available.

^b^EEG: electroencephalography.

^c^ECG: electrocardiography.

^d^PPG: photoplethysmography.

**Table 4:** Target health outcomes by sensor type and biomarkers (miscellaneous).

		Target health outcomes, n (%)			Total (row; N=51), n (%)
		Combination (n=8)	Other (n=6)		
**Sensor types**					
	Electrical	1 (12)	1 (17)	19 (37)	
	Photo	1 (12)	1 (17)	9 (18)	
	Mechanical	—^a^	1 (17)	5 (10)	
	Thermal	—	2 (33)	3 (6)	
	Acoustic	1 (12)	—	1 (2)	
	Combination	5 (62)	1 (17)	14 (27)	
**Biomarkers**					
	EEG^b^	—	—	11 (22)	
	Body movements	1 (12)	2 (33)	6 (12)	
	Body temperature	1 (12)	3 (50)	5 (10)	
	ECG^c^	—	—	4 (8)	
	Acceleration stress	—	—	4 (8)	
	PPG^d^	1 (12)	—	3 (6)	
	Combination	4 (50)	1 (17)	13 (25)	
	Other	1 (12)	—	5 (10)	
Total (column; N=51)		8 (16)	6 (12)	51 (100)	

^a^Not available.

^b^EEG: electroencephalography.

^c^ECG: electrocardiography.

^d^PPG: photoplethysmography.

### Applications to Prevention

Table 5 presents the cross-tabulated data for target health outcomes, preventive stage, biomarkers, and sensor types. The first section of the table cross-tabulates health or disease status and preventive stage. Some studies of dietary status [32-39], brain status [42,43], respiration [20], and other outcomes [72,75] were classified as primary prevention. Other studies of brain status [40,41,44,45,78], CVD [46-50], CNS disease [51-54,79], heart status [59-61], respiration [62], diet monitoring to help patients with gastric cancer [19], sleep disorders [63,64], and combinations of health status with other outcomes [71,73] were classified as secondary prevention; this category was the largest. Earable devices were used for tertiary prevention in 8% (4/51) of the studies and were applied to support people with hand disability by sensing their tongue motion to operate a portable audio player [18]. Gait classification provided information related to Parkinson disease [68], and human body potentials provided information related to facial expressions in patients with locked-in syndrome [74] and for the stimulation treatment of patients with Parkinson disease [76].

Biomarkers used in the primary prevention studies included body movements [20,33,75], EEG [43], body temperature [72], PPG [32], and combined biomarkers (eg, PPG plus bioacoustics, with or without vibration or air pressure measurements; or including EEG, electro-oculography, and EMG [34-36,38,42]). Biomarkers used in secondary prevention studies included EEG [40,41,44,45,51-54,63,78], body temperature [22,64,71,73], ECG [49,50,59], PPG [48,60,69], body movement [21], oxygen saturation [62], EPWs [47], and combinations of biomarkers (eg, EMG and ear canal shape to estimate occlusal force [39]; ECG and lactate level in relation to head acceleration [66]; body movements, temperature, and HRV [68]; EEG, acoustic signals, and MPG [65]; ECG combined with ballistocardiography and PPG [46]; EEG and ECG [70]; and PPG and air pressure [61]). The biomarkers used in the tertiary prevention studies were body movements and ear canal shape [18,19], caloric vestibular stimulation [79], and combined biomarkers including human body potentials [74].

Sensor types according to prevention stage are detailed in Table 5. Sensors used in the primary prevention studies included photosensors [20,32,33], electric sensors [37,42,43], thermal sensors [72], and mechanical sensors [75]. Photosensors and acoustic sensors were also used in combination with no mechanical sensor [34,36]. Mechanical, acoustic, electrical, and pressure sensors were used in 2% (1/51) of the studies [35,38]. Sensors used in the secondary prevention studies were electric [40,41,44,45,47,49-54,59,63,70,78], mechanical [55-58], photo [48,60,62,69], thermal [73], acoustic [67], and combined [21,22,39,46,61,64-66,68,71]. Sensors used in the tertiary prevention studies were photosensors [18,19], electrical sensors [74], and thermal sensors [76].

The sensor positions used in the primary prevention studies were the ear canal [20,33,38,42,43,72], behind the ear [35,37], the concha [34], the earlobe [32], the inner ear [75], and multiple positions [36]. The sensor positions in the secondary prevention studies were the ear canal [21,22,40,44,45,47,50,58,60-65,67,68,71], behind the ear [51,54-57,59], around the ear [41,66,70], the earlobe [48,69], and multiple positions [39,46,49,52,53,78]. The sensor positions in the tertiary prevention studies were the ear canal [18,19,74] and multiple positions [76].

**Table 5:** Outcomes measured using the earable devices by prevention stage.

			Prevention stage, n (%)							Total (row; N=51), n (%)
			Primary (n=12)		Secondary (n=35)		Tertiary (n=4)			
**Target health outcomes**										
	Diet-related	7 (58)		1 (3)		1 (25)		9 (18)		
	Brain status	2 (17)		5 (14)		—^a^		7 (14)		
	Cardiovascular disease	—		5 (14)		—		5 (10)		
	Central nervous system disease	—		4 (11)		1 (25)		5 (10)		
	Head injury	—		4 (11)		—		4 (8)		
	Heart status	—		3 (9)		—		3 (6)		
	Respiration	1 (8)		1 (3)		—		2 (4)		
	Sleep disorder	—		2 (6)		—		2 (4)		
	Combination	—		8 (23)		—		8 (16)		
	Other	2 (17)		2 (6)		2 (50)		6 (12)		
**Biomarkers**										
	EEG^b^	1 (8)		10 (29)		—		11 (22)		
	Body movements	3 (25)		1 (3)		2 (50)		6 (12)		
	Body temperature	1 (8)		4 (11)		—		5 (10)		
	PPG^c^	1 (8)		3 (9)		—		4 (8)		
	Acceleration stress	—		4 (11)		—		4 (8)		
	ECG^d^	—		3 (9)		—		3 (6)		
	Combination	5 (42)		7 (20)		1 (25)		13 (25)		
	Other	1 (8)		3 (9)		1 (25)		5 (10)		
**Sensor type**										
	Electric	3 (25)		15 (43)		1 (25)		19 (37)		
	Photo	3 (25)		4 (11)		2 (50)		9 (18)		
	Mechanical	1 (8)		4 (11)		—		5 (10)		
	Thermal	1 (8)		1 (3)		1 (25)		3 (6)		
	Acoustic	—		1 (3)		—		1 (2)		
	Combination	4 (33)		10 (29)		—		14 (27)		
Total (column; N=51)			12 (24)		35 (69)		4 (8)		51 (100)	

^a^Not available.

^b^EEG: electroencephalography.

^c^PPG: photoplethysmography.

^d^ECG: electrocardiography.

## Discussion

### Summary of Review Results

We assessed the health-related indicators measured using earable devices and the utility of these devices for public health. Earable devices can measure various health and disease states related to morbidity and, thus, can be used to propose solutions for health care systems in real time [80]. The detection of various health-related indicators has improved since earable devices were first introduced. Most of the earable devices in our review measured single health outcomes using 1 biomarker and sensor. However, several studies (3/51, 6%) assessed multiple health outcomes using combinations of biomarkers and sensors.

Most of the health outcomes assessed by the studies in this review were related to diet (assessed through mastication monitoring), brain status, CNS diseases, heart conditions, CVDs, head injury, respiration, and sleep disorders (monitored in real time). Biomarkers of health outcomes and conditions included EEG, muscle and body movements, body temperature, PPG, ECG, and acceleration stress. Electrical sensors were used to obtain physiological information and convert it into electrical signals, including EEG and ECG. Photosensors were mostly used to detect PPG to monitor heart and dietary status. In addition, body movements were detected using used photosensors to assess physical disability and diet and respiration quality. Mechanical sensors were mostly used to monitor head injuries based on head location and acceleration. Thermal sensors were used to monitor body temperature and aid in the treatment of Parkinson disease. Regarding the positions of the sensors, nearly half of the devices were inserted into the ear canal. Sensors attached behind the ear obtained EEG signals for head injury management or head acceleration and location information to detect CNS disease. PPG data were obtained through sensors on the earlobe; these data were relevant to CVDs and mastication.

The preventive applications of earable devices are classified according to the characteristics of target health outcomes and the biomarkers used for detection. In primary prevention studies, healthy diet was the most common outcome measure based on mastication or occlusal force determined using photosensor signals reflecting changes in ear canal shape. Most studies were related to secondary prevention, indicating that a constant trend in earable device application is risk factor monitoring. Biomarkers such as EEG, ECG, PPG, body temperature, and acceleration stress were monitored as target health outcomes. EEG was used to provide evidence of CNS disease, sleep disorders, and stress in cases of symptoms such as seizures, sleep disturbance, and negative emotions. The risk of CVD was detected using ECG and PPG sensors, which captured heart condition indicators such as the HR and pulse rate to identify prodromal conditions (eg, atrial fibrillation, ventricular bigeminy, hypertension, and hemodialysis). Physical impacts after head injuries such as concussion were monitored using acceleration stress data from mechanical sensors. Combined sensor applications enabled multi-disease monitoring for research, including that on common health problems (eg, fever, fatigue, insomnia, and depression), diseases (eg, CNS diseases, CVDs, metabolic disorders, and mental disorders), and motion. For tertiary prevention, Taniguchi et al [19] explored the provision of dietary support via earable devices detecting ear canal shape and occlusal force for patients with gastric cancer. Wilkinson et al [76] evaluated the effectiveness of caloric vestibular stimulation for patients with Parkinson disease. Burgos et al [68] developed earable devices to target physical activity in real life by detecting gait and HRV, which can be applied to older adults, individuals with obesity, and patients with disabilities.

### Key Messages of the Review

Earable devices can obtain ECG, PPG, glucose, body temperature, acceleration, and pressure data as biomarkers of health and disease status. A previous systematic review identified four domains: health and safety monitoring, chronic disease management, disease diagnosis and treatment, and rehabilitation [9]. Biomarkers of health conditions and diseases can be obtained through earable devices to aid prevention and management.

The application of the IoT to personal health management via earable sensors promotes secondary prevention through real-time health status monitoring [9,81]. In the context of primary prevention, earable devices can improve health behaviors. In terms of tertiary prevention, wearable devices can support body parts functioning with difficulties because of physical disabilities [81].

The mobile health platform is used to engage the public in research, for example, on devices developed for supporting various body parts. SMS text messages and smartphone apps are commonly used for public engagement [9,82]. Wearables for the health and medical field are promising but still have shortcomings in terms of user-friendliness, security and privacy, and technical issues [83]. Earable devices are an alternative platform that may overcome these shortcomings. However, more research and development are needed [3].

**Figure 2:**
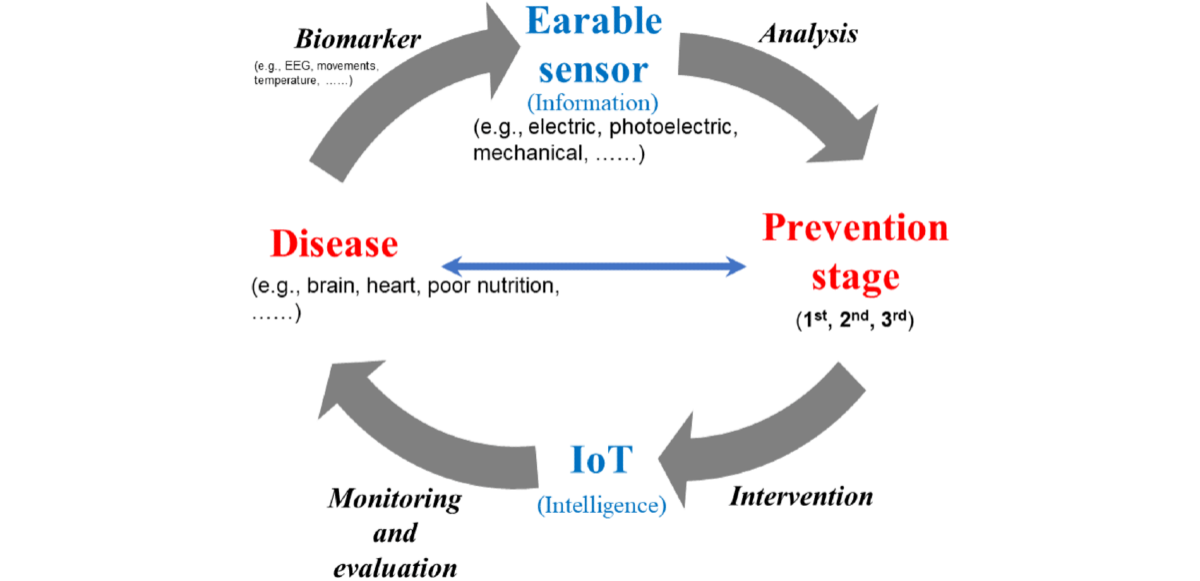
Health care and prevention framework for earable devices. EEG: electroencephalography; IoT: Internet of Things.

Earable devices with built-in sensing technology can accurately transmit digital health care information for use for preventive strategies. A health care and prevention framework was devised based on keywords extracted from earable device studies (Figure 2). The horizontal axis (red) represents health care access, including target health conditions and prevention stages that can potentially improve health outcomes. Health care information derived from earable devices is useful for all preventive processes related to health improvement in personal care, monitoring and diagnosis, and treatment and rehabilitation. The vertical axis (blue) represents technology access, which ranges from information collection using earable sensors to the integration of the collected information with broader contextual knowledge to aid the design and implementation of appropriate interventions at the appropriate time. The diagonal arrow (black) indicates the activity at each stage. Information about a disease is transmitted in the form of a biomarker and analyzed and classified according to the prevention stage. Health care interventions based on IoT assistance and technology are implemented to monitor and assess conditions of target health outcomes.

### Earable Devices in the IoT Era

In the IoT era, devices for protecting against excessive noise, hearing aids, and an in-ear EEG brain-computer interface have been developed. Physiological and electrophysiological data, ear canal deformation, dynamic measurements, medical condition management, and biosignal data can be obtained or achieved through in-ear devices. Communication can also be enhanced via stimulation with electricity and light, energy harvesting, and noise cancellation [84]. Earable devices based on IoT demonstrate improved data collection and processing accuracy, timely alarm-warning signals, and high usability and consumer acceptability [9,81].

Improvements in earable devices and their applications compared with older devices were discussed in some of the reviewed articles (18/51, 35%). Emotion monitoring has been reported using a headset and Bluetooth device for use in the home and remotely, respectively [44]. ECG data from athletes, firefighters, and pilots have been collected via a mono-earphone compatible with a smartphone [73]. EEG [41,45,52], body movements [18-21,75], and core body temperature [22,64,71,72] data can also be obtained through smartphone apps in real time using in-house software and through tablets. A study described a web-based personal coaching system based on sensors and smartphone apps (the “SPLENDID” system) [36]. Another study showed that earable device–based sleep quality monitoring systems can improve sleep quality in the community setting [85]. Suggested functions for earable devices in the IoT era include data processing for personal health care. Accumulated data demonstrate the stability of data sets collected through cloud servers available only to the individual concerned and related users [9,80].

### Applications of Earable Devices for Disease Prevention

The current focus with respect to wearable device–based personal health care is on improving diagnostics and health behaviors. Additional considerations include gathering microenvironmental data relevant to disease risk and merging multiple strata of health care into a single integrated form [9,86]. The National Health Plan 2030 in Korea provides a systematic framework for the prevention of various health risks to improve the lifelong health of individuals. In particular, the plan includes a goal to “develop health-friendly environments” with a focus on the “application of innovative information technology” [87]. The development of useful wearable devices is expected to facilitate the achievement of the National Health Plan 2030 goals.

Regarding primary prevention in the context of eHealth, interventions and guidelines pertaining to diet, mental workload, ovulation, and respiration are needed. Strategies emphasizing the monitoring of health status can improve health. As wearable devices are capable of real-time monitoring, intervention before disease manifests is possible [4,10,81]. Smartphone apps using diary or daily chart functions can be used to track dietary behaviors, and earable devices can obtain chewing and food intake data automatically [32]. Mental workload measurement is also valuable to reduce the likelihood of occupational accidents via alerts [43]. Ovulation and respiration data can also be obtained instantaneously, facilitating pregnancy planning and meditation, respectively [20,72].

Regarding secondary prevention, vital signs can be monitored using earable devices to aid disease diagnosis. Brain monitoring (EEG) can help diagnose CNS diseases, and cardiac monitoring (ECG, PPG, and other biomarkers) can help detect CVDs using well-established display options. Body temperature and body movement can also be measured for secondary preventive purposes. Earable devices have been validated as a replacement for traditional biomarker detection methods. A study in this review using caloric vestibular stimulation as a treatment for Parkinson disease was classified as secondary prevention and was the only study to use this treatment [76].

The use of earable devices for tertiary prevention may be improved, but 2% (1/51) of the studies suggested that hands-free (ear-worn) devices could be used in the future for motion detection in individuals with disabilities to facilitate self-management [18].

The effective use of earable devices involves when and how the devices should be used. For primary prevention, earable devices can be used to monitor healthy lifestyle factors such as physical activity and eating habits. Physical activity monitoring using earable devices should encompass leisure, work, and travel times, similar to smartwatch device monitoring. The use of earable devices to monitor eating habits may provide unique advantages over smartwatch use [38]. The use of these devices for secondary prevention can be facilitated by collaboration with clinicians who assess the need for continuous monitoring of specific biomarkers such as EEG, ECG, or acceleration stress. Several studies have focused on the use of earable devices for the monitoring of seizures, brain injuries, sleep disorders, arrhythmias, and myocardial infarction [45,49-51,53-58,63]. Clinicians can determine the specific timing of monitoring according to patients’ needs.

The collection and analysis of data from earable devices typically require smartphone connection to an IoT platform that includes a central database system. The amount of information collected is substantially increased by the use of multimodal sensors. Furthermore, data collection can be expanded through the simultaneous use of earable devices, smartwatches, and smartphones. The development of IoT technology for the real-time analysis of data from various devices is expected. However, data from different devices are currently downloaded separately and used in combined analyses. User-friendly smartphone apps that summarize earable device data will help users plan their health management [38,72].

The capacity of earable devices with IoT platforms for continuous monitoring and in-depth analysis is expected to shift the focus of prevention toward active health promotion. Thus, we propose improved definitions of prevention linked to the use of earable devices (Textbox 2).

Prevention based on individual efforts is not effective. Public health services and policies should be directed toward the empowerment of individuals through the provision of supportive tools and environments.

**Improved definitions of prevention linked to the use of earable devices.**

**Improved definitions of prevention**

- Primary prevention: intervention before the disease process begins through the avoidance of health risk factors and practice of health-promoting behaviors with continuous monitoring of health status to maintain motivation
- Secondary prevention: screening for early diagnosis and prediction of the risk of disease occurrence via intelligent analysis of monitoring data
- Tertiary prevention: attenuation of disease progression via appropriate treatment and rehabilitation and improvement of the quality of life via the enhancement of self-management ability with the assistance of a smart care platform

### Role of IoT in Earable Devices for Monitoring Health Outcomes

IoT-based earable devices will make microlevel monitoring possible and, thus, improve health care through their interactions with other, nonwearable devices. Earable devices based on IoT will facilitate personal health care and also help physicians [88,89]. These devices will allow the health status of patients to be continually tracked, particularly older adult patients who live alone; if any changes in health status occur, the devices can alert family members or health care providers immediately. In addition to the monitoring capabilities of earable devices based on the IoT, they can help physicians manage their patients’ treatments more effectively. They can also help health care facilities function in an orderly manner [89,90] as the devices can be tracked in real time within hospitals. Moreover, they can be used to monitor environmental conditions and the hygiene, body temperature, and location of medical staff [88,91].

New technologies allow for the remote treatment of patients [85,92,93]. Mobile apps can provide guidance at the population level, including on medications and habits, and effective strategies for mitigating the risk of stroke and CVD [93,94].

### Validity of Earable Device Measurements

Most studies of earable devices have determined that the use of biomarkers in experimental or real-environment settings is sufficiently valid to replace conventional standards of measurement. Patient-independent and patient-specific models of EEG-based detection using a behind-the-ear device compared with a professional’s visual seizure annotation and the use of a data-driven algorithm, respectively, showed that device detection had 65.7% sensitivity and 94.4% specificity compared with visual recognition. Similar results were obtained in comparison with the automated algorithm; the patient-independent model indicated that device detection had 64.1% sensitivity and 2.8 false-positive detections per 24 hours, and the patient-specific model yielded values of 69.1% sensitivity and 0.49 false-positive detections per 24 hours. Thus, the patient-specific model confirmed the best performance of behind-the-ear EEG detection [54]. The performance of body movement detection with an earable device was assessed using chew counts in gum-chewing and almond-eating tests; the device showed 95.8% precision, better than recall (93.7%), reflecting accurate counting and the ability to distinguish chewing from other activities [19]. Body temperature detection by an earable device for ovulation detection and prediction in 34 participants was evaluated and based on the relative distance of the estimated from the nearest self-reported ovulation day, the device showed improved detection accuracy, with 92.3% sensitivity and 23.1% to 31.6% greater predictive power [72]. PPG-based monitoring for CVD detection was evaluated using parameter values in a learning data set, and the lowest level of sensitivity and specificity was 90.9% [48]. Acceleration stress measurement using an earable device, used primarily in head impact monitoring, was evaluated in youth soccer players. Random and systematic errors were calculated, and areas under the curve were used to confirm the device’s capacity in on-field settings. Cutoff values for prediction were 100% in structured training sessions and 65% in regular soccer sessions, although improvement is needed because of the overestimation of impact exposure and random error [58].

### Implications

A considerable number of studies included in this review (34/51, 66.7%) focused on single-target health outcomes with the use of single sensors, although some were concerned with multiple health outcomes and involved the use of several sensors. The use of multiple sensors increases the accuracy of activity analysis, and the collection of physiological data in real time is useful for the exploration of mental and physical health [95]. In some studies (6/51, 12%), various sensors (eg, air pressure, piezoelectric strain, and electric sensors) were combined in single devices to detect a single target health outcome (ie, mastication related to healthy diet). The sensors used EMG, ear canal pressure, and muscle movement biomarkers to differentiate food types. The accuracy of the indicators of chewing strength remains uncertain [38]. Other sensor combinations, including photo, mechanical, and electric sensors, have also been used in single devices. A device that captures signals of body temperature, acceleration stress, and HRV simultaneously provides information about the risk of heart disease and gait disturbances with reliable accuracy and no wearer discomfort. Although the device cannot easily distinguish between walking and running, elaborate calibration effectively provides this capacity [68]. Future research should examine the feasibility of combining sensors and processing of the obtained data.

In the studies included in this review, the sensor type and health outcomes of interest differed according to sensor location within the ear; devices placed in the ear canal were used in almost half of the studies (26/51, 51%). However, ear sensors can have problems related to wearability, small size, battery life, and real-time signal processing [96]. Traditionally, biomarkers have not been measured in the ear but, with further advances in technology, the ear will become more attractive as a location for measurement devices relative to other body parts.

Digital therapeutics is an emerging field of application that involves the use of digital technology for health care [97]. It has been growing steadily with the development of several new programs and apps [98,99]. Interventions targeting obesity and dietary habits have been used for technology-supported and mobile device–based smart group care, the restriction of eating times, and digitally-assisted cognitive behavioral therapy. Wearables were also used in these programs [97,100]. Digital health care services and the development of wearable technology and IoT services will play important roles in the future as parts of public health services (ie, DPH). After the COVID-19 pandemic, the demand for DPH tools such as tracing apps, chatbots providing COVID-19 information, and digital mental health support services has increased sharply. Large-scale data accessibility is expected to facilitate sustainable DPH [28]. Future earable IoT systems have potential uses in DPH because of their advantages for real-time monitoring and analysis.

Wearable technology is widely used in health care to prevent, diagnose, manage, and treat conditions and for patient rehabilitation. IoT could contribute to the development of smart homes [101], smart cities [102], and smart governance [91]. This review suggests the possibility for the further development of earable devices to obtain evidence-based data that inform policies and regulations for smart homes and smart cities and for the provision of medical services for all patient populations [81,91,101,102].

### Limitations

This review has some limitations. Even though available standardized terms from 3 databases were included, our search strategy may have missed some articles. This review was exploratory in nature and included a wide range of study designs, leading to the possibility of heterogeneity. By the research design of this review, the research settings and assessment of risk of bias in individual studies were not described in detail during extraction of the characteristics of earable devices. Finally, the subjectivity in data collection might have remained even if a validation process in data extraction and collection was used to mitigate the bias.

### Conclusions

Health-related indicators and biomarkers detected using earable devices can be used to monitor health outcomes. Brain status, healthy diet status, and CVD were the most frequently measured outcomes. Combinations of targeted biomarkers were collected using several sensors in some studies. Earable devices can be used for secondary prevention by monitoring health or disease status and also have potential for primary prevention. However, use for tertiary prevention was limited and particularly called for more research. Earable devices can be connected to smartphones or tablets through cloud servers for guaranteed accessibility and compatibility of continuous health monitoring data.

## Appendix

Multimedia Appendix 1 A model for the relationships among wearables, earables, and hearables.

Multimedia Appendix 2 Systematic review methodology.

Multimedia Appendix 3 Data abstraction from selected articles.

Multimedia Appendix 4 Key properties of earable devices and health-related indicators.

## Abbreviations

AMSTAR-2: A Measurement Tool to Assess Systematic Reviews
CNS: central nervous system
CVD: cardiovascular disease
DPH: digital public health
ECG: electrocardiography
EEG: electroencephalography
EMG: electromyography
EPW: ear pulse wave
HR: heart rate
HRV: heart rate variability
IoT: Internet of Things
MPG: mechanical plethysmography
PPG: photoplethysmography
PRISMA: Preferred Reporting Items for Systematic Reviews and Meta-Analyses
