# Supplementary figures and images for: Health-Related Indicators Measured Using Earable Devices: Systematic Review
Source: JMIR Mhealth Uhealth. 2022 Nov 15;10(11):e36696. doi: 10.2196/36696 (PMC9709679; doi:10.2196/36696)

## Slide 1
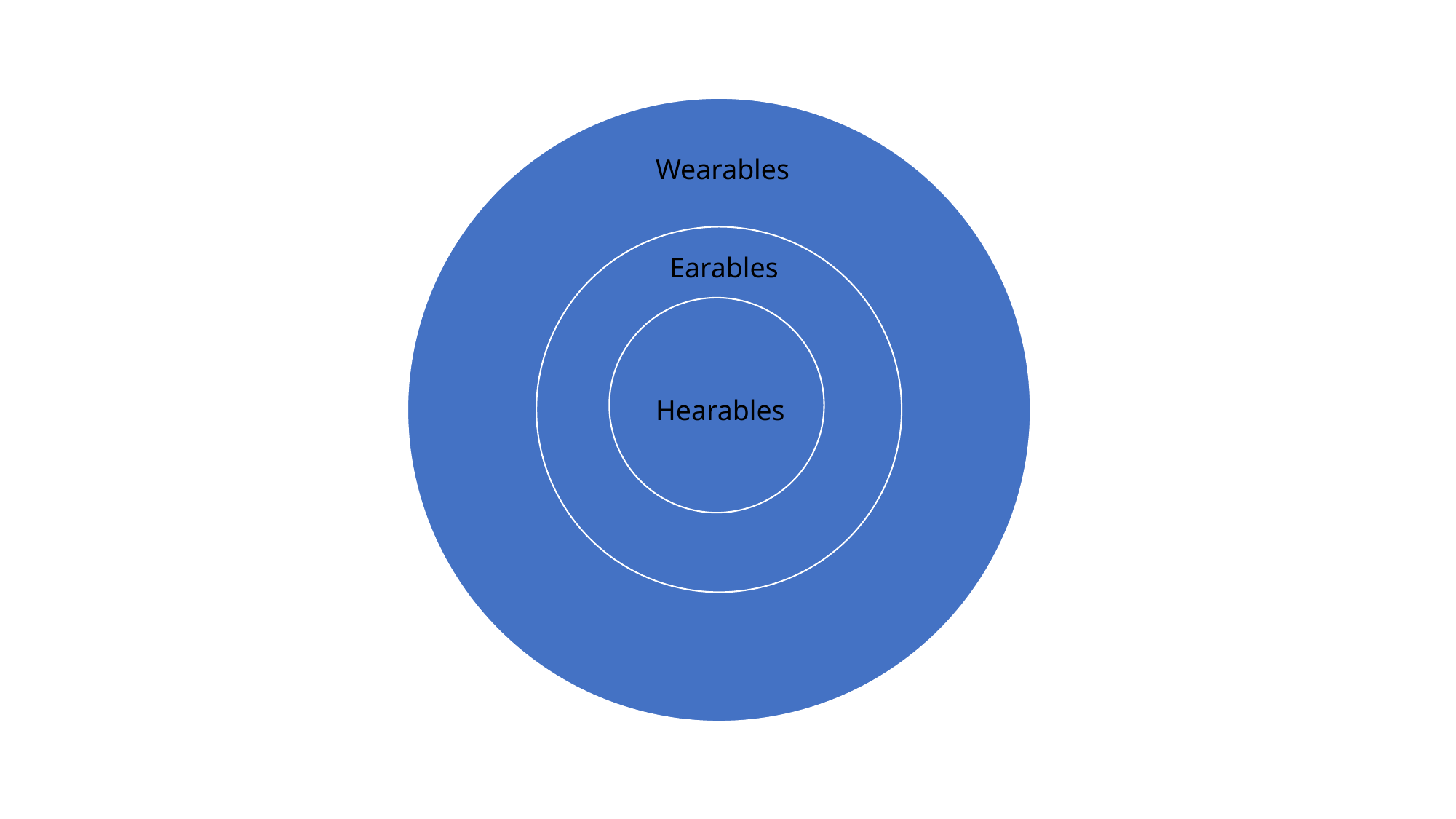

Wearables
Earables
Hearables

Supplement: Multimedia Appendix 1 [file mhealth_v10i11e36696_app1.pptx]
