# Supplementary material for: Health-Related Indicators Measured Using Earable Devices: Systematic Review
Source: JMIR Mhealth Uhealth. 2022 Nov 15;10(11):e36696. doi: 10.2196/36696 (PMC9709679; doi:10.2196/36696)
Supplement: Multimedia Appendix 2 [file mhealth_v10i11e36696_app2.docx]

**Multimedia Appendix 2. Systematic review methodology.**

Supplementary table 1. Search strategy.

|  | Keyword | No. of hits | Search date |
| --- | --- | --- | --- |
| **PubMed** | ((("ear"[MeSH Terms] OR "ear"[All Fields]) AND ("wearable electronic devices"[MeSH Terms] OR "wearability"[All Fields] OR "wearable"[All Fields] OR "wearables"[All Fields])) OR "earable*"[All Fields] OR "hearable*"[All Fields]) AND (2015:2020[pdat]) | 881 | 12. 04. 2021 |
| **Web of Science** | ALL=((ear AND (wearable electronic devices OR wearability OR wearable OR wearables)) OR earable* OR hearable*) AND FPY=(2015-2020) | 170 | 12. 04. 2021 |
| **Embase** | (('ear'/exp OR ear) AND ('wearable*' OR 'wearable computer' OR 'wearable sensor')) OR earable OR 'hearable*' AND (2015:py OR 2016:py OR 2017:py OR 2018:py OR 2019:py OR 2020:py) | 139 | 12. 04. 2021 |
| **Total** | | 1,190 |  |

Supplementary table 2. Inclusion/Exclusion criteria.

| **Inclusion criteria** | **Exclusion criteria** |
| --- | --- |
| Study articles written in English | Study articles written in non-English |
| Full-text articles | Study articles including only abstract |
| Study articles including original research | Study articles not mainly constructed with original research (e.g., Reviews, editorials, letters, supplementary articles) |
| Study articles are including human participants | Study articles not targeting humans |
| Study articles mainly covers non-auditory functions of the body  (e.g., physiological effects, physical impact, related diseases) | Study articles mainly covers hearing functions and diseases |

Supplementary table 3. Quality Assessment of the Systematic Review (AMSTAR 2).

AMSTAR 2: a critical appraisal tool for systematic reviews that include randomised or non-randomised studies of healthcare interventions, or both

| **AMSTAR criteria** | **Our manuscript** |
| --- | --- |
| 1. Did the research questions and inclusion criteria for the review include the components of PICO? | No |
| 1. Did the report of the review contain an explicit statement that the review methods were established prior to the conduct of the review and did the report justify any significant deviations from the protocol? | Partial Yes  (review questions, search strategy, inclusion/exclusion criteria, plan for investigating causes of heterogeneity, justification for any deviations from the protocol) |
| 1. Did the review authors explain their selection of the study designs for inclusion in the review? | Yes  (OR Explanation for including both RCTs and NRSI) |
| 1. Did the review authors use a comprehensive literature search strategy? | Yes |
| 1. Did the review authors perform study selection in duplicate? | Yes  (at least two reviewers independently agreed on selection of eligible studies and achieved consensus on which studies to include) |
| 1. Did the review authors perform data extraction in duplicate? | Yes  (at least two reviewers achieved consensus on which data to extract from included studies) |
| 1. Did the review authors provide a list of excluded studies and justify the exclusions? | Yes |
| 1. Did the review authors describe the included studies in adequate detail? | No |

Supplementary table 3. Quality Assessment of the Systematic Review (AMSTAR 2) (continued).

| **AMSTAR criteria** | **Our manuscript** |
| --- | --- |
| 1. Did the review authors use a satisfactory technique for assessing the risk of bias (RoB) in individual studies that were included in the review? | No |
| 1. Did the review authors report on the sources of funding for the studies included in the review? | Yes |
| 1. If meta-analysis was performed did the review authors use appropriate methods for statistical combination of results? RCTs | No meta-analysis conducted |
| 1. If meta-analysis was performed, did the review authors assess the potential impact of RoB in individual studies on the results of the meta-analysis or other evidence synthesis? | No meta-analysis conducted |
| 1. Did the review authors account for RoB in individual studies when interpreting/ discussing the results of the review? | Yes  (NRSI were included the review provided a discussion of the likely impact of RoB on the results ) |
| 1. Did the review authors provide a satisfactory explanation for, and discussion of, any heterogeneity observed in the results of the review? | No  (No meta-analysis conducted) |
| 1. If they performed quantitative synthesis did the review authors carry out an adequate investigation of publication bias (small study bias) and discuss its likely impact on the results of the review? | No meta-analysis conducted |
| 1. Did the review authors report any potential sources of conflict of interest, including any funding they received for conducting the review? | Yes  (The authors reported no competing interests) |
