# Supplementary material for: Health-Related Indicators Measured Using Earable Devices: Systematic Review
Source: JMIR Mhealth Uhealth. 2022 Nov 15;10(11):e36696. doi: 10.2196/36696 (PMC9709679; doi:10.2196/36696)
Supplement: Multimedia Appendix 3 [file mhealth_v10i11e36696_app3.docx]

**Multimedia Appendix 3. Data abstraction from selected articles.**

Supplementary Table 4. The list of the identified relevant research articles from the three selected digital libraries.

| Author | Publication Year | Target health outcomes | Biomarker | Sensor type | Sensor position | Prevention stage |
| --- | --- | --- | --- | --- | --- | --- |
| He et al [46] | 2015 | CVD (heart rate, PEP, SV, CO, PTT) | Combined (ECG, BCG, PPG) | Combined (electric, mechanical, photo) | Multiple (in-ear. near the ear, behind the ear) | Secondary |
| Park et al [47] | 2015 | CVD (heart rate, blood pressure) | Others (EPW) | Electric (piezoelectric) | Ear canal | Secondary |
| Goverdovsky et al [40] | 2016 | Brain related (brain activity) | EEG | Electric | Ear canal | Secondary |
| Looney et al [63] | 2016 | Sleep disorder (obstructive sleep apnea) | EEG | Electric | Ear canal | Secondary |
| Papapanagiotou et al [32] | 2016 | Diet-related (mastication) | PPG | Photo | Earlobe  (outer earlobe) | Primary |
| Bedri et al [35] | 2017 | Diet-related (hypercholesterolemia, obesity, diabetes) | Combined (shape of ear canal, muscle movement, swallowing sound) | Combined (mechanical, acoustic) | Behind the ear | Primary |
| Bleichner; Debener [41] | 2017 | Brain related (Brain) | EEG | Electric | Around the ear | Secondary |
| Conroy et al [48] | 2017 | CVD (atrial, fibrillation, arrhythmia) | PPG | Photo | Earlobe | Secondary |
| Goverdovsky et al [65] | 2017 | Combined (brain, cardiac, respiratory function) | Combined (EEG, breathing sound, MPG) | Combined (mechanical, electric) | Ear canal | Secondary |
| Gu et al [51] | 2017 | CNS disease (seizure, epilepsy) | EEG | Electric | Behind the ear | Secondary |

Supplementary Table 4. The list of the identified relevant research articles from the three selected digital libraries (continued).

| Author | Publication Year | Target health outcomes | Biomarker | Sensor type | Sensor position | Prevention stage |
| --- | --- | --- | --- | --- | --- | --- |
| Matthies et al [74] | 2017 | Others (facial expression) | Combined  (human body potential) | Electric | Ear canal | Tertiary |
| Ota et al [22] | 2017 | Combined (fever, fatigue, insomnia, depression) | Body temperature | Combined (electric, thermal) | Ear canal | Secondary |
| Papapanagiotou et al [34] | 2017 | Diet related (mastication) | Combined (PPG, bioacoustic) | Combined (photo, acoustic) | Ear concha | Primary |
| Taniguchi et al [33] | 2017 | Diet related (dietary support) | Body movement (shape of ear canal) | Photo | Ear canal | Primary |
| von Rosenberg et al [49] | 2017 | CVD (myocardial infarction, atrial fibrillation) | ECG | Electric | Multiple (head-surface, ear canal) | Secondary |
| Zibrandtsen et al [52] | 2017 | CNS disease (ictal and interictal abnormalities, temporal lobe epilepsy) | EEG | Electric | Multiple (ear canal, concha) | Secondary |
| Chaglla et al [71] | 2018 | Others (hypothermia, hyperthermia, heat stroke) | Body temperature | Combined (electric, thermal) | Ear canal | Secondary |
| Hecimovich et al [57] | 2018 | Head injury (concussion) | Acceleration stress (peak linear-rotational acceleration, impact location) | Mechanical | Behind the ear | Secondary |
| Hurst et al [55] | 2018 | Head injury (concussion, mild traumatic brain injuries) | Acceleration stress (magnitude of translational and rotational neck acceleration) | Mechanical | Behind the ear | Secondary |

Supplementary Table 4. The list of the identified relevant research articles from the three selected digital libraries (continued).

| Author | Publication Year | Target health outcomes | Biomarker | Sensor type | Sensor position | Prevention stage |
| --- | --- | --- | --- | --- | --- | --- |
| Jacob et al [59] | 2018 | Heart related (heart rate, frequency) | ECG | Electric | Behind the ear | Secondary |
| King et al [56] | 2018 | Head injury  (concussion) | Acceleration stress (peak linear-rotational acceleration, impact location) | Mechanical | Behind the ear | Secondary |
| Martin; Voix [67] | 2018 | Combined (heart rate, breathing rate) | Others (in-ear sound, mouth breathing, nasal breathing) | Acoustic | Ear canal | Secondary |
| Nguyen et al [42] | 2018 | Brain related (sleep quality) | Combined (EEG, EOG, EMG) | Electric | Ear canal | Primary |
| Taniguchi et al [18] | 2018 | Others (physical disabilities) | Body movement (shape of ear canal) | Photo | Ear canal | Tertiary |
| Taniguchi et al [19] | 2018 | Diet related (gastric cancer) | Body movement (shape of ear canal, occlusal force) | Photo | Ear canal | Tertiary |
| Taniguchi; Nishikawa [20] | 2018 | Respiration (respiratory rate) | Body movement (shape of ear canal) | Photo | Ear canal | Primary |
| Taniguchi; Nishikawa [21] | 2018 | Combined (respiration, posture) | Body movement (shape and shaking of ear canal) | Combined (photo, mechanical) | Ear canal | Secondary |
| van den Boer et al [36] | 2018 | Diet related (obesity, overweight) | Combined (PPG, bioacoustics, vibration) | Combined (photo, acoustic, mechanical) | Multiple (in ear, around the ear) | Primary |
| Zhang; Amft [37] | 2018 | Diet related (dietary behavior) | Others (EMG) | Electric | Behind the ear | Primary |
| Ahn et al [70] | 2019 | Combined (chronic stress, cognitive dysfunction) | Combined (EEG, ECG) | Electric | Around the ear | Secondary |

Supplementary Table 4. The list of the identified relevant research articles from the three selected digital libraries (continued).

| Author | Publication Year | Target health outcomes | Biomarker | Sensor type | Sensor position | Prevention stage |
| --- | --- | --- | --- | --- | --- | --- |
| Athavipach et al [44] | 2019 | Brain related (emotion) | EEG | Electric | Ear canal | Secondary |
| Bui et al [61] | 2019 | Heart related (heart failure, hypertension, hemodialysis) | Combined (PPG, air pressure) | Combined (photo, mechanical) | Ear canal | Secondary |
| Gil et al [66] | 2019 | Combined (cardiovascular condition, sweat, motion) | Combined (ECG, PH-lactate, head acceleration) | Combined (electric, amperometric, potentiometric, mechanical) | Around the ear | Secondary |
| Hammour et al [50] | 2019 | CVD (arrhythmias, ventricular bigeminy) | ECG | Electric | Ear canal | Secondary |
| Hoelzemann et al [75] | 2019 | Others (physical activity) | Body movement (acceleration) | Mechanical | Inner ear | Primary |
| Kappel et al [44] | 2019 | Brain related | EEG | Electric | Multiple (ear canal, concha) | Secondary |
| Ko et al [64] | 2019 | Sleep disorder (sleep disturbance, affective disorders) | Body temperature | Combined (electric, thermal) | Ear canal | Secondary |
| Kuatsjah et al [43] | 2019 | Brain related (mental workload) | EEG | Electric | Ear canal | Primary |
| Kurosawa et al [39] | 2019 | Diet related (gastric cancer) | Combined (shape of ear canal, EMG, occlusal force) | Combined (photo, electric, mechanical) | Multiple (ear canal, masseter muscle, oral cavity) | Secondary |
| Passler et al [60] | 2019 | Heart related (heart rate, pulse) | PPG | Photo | Ear canal | Secondary |
| Sandmo et al [58] | 2019 | Head injury (sub-concussive head impacts) | Acceleration stress (peak linear-rotational acceleration, velocity) | Mechanical | Ear canal | Secondary |

Supplementary Table 4. The list of the identified relevant research articles from the three selected digital libraries (continued).

| Author | Publication Year | Target health outcomes | Biomarker | Sensor type | Sensor position | Prevention stage |
| --- | --- | --- | --- | --- | --- | --- |
| Wilkinson et al [76] | 2019 | CNS disease (Parkinson’s disease) | Others (caloric vestibular stimulation) | Thermal | Multiple (ear canal, concha) | Tertiary |
| Burgos et al [68] | 2020 | Combined (heart attack, gait) | Combined (inertial linear and gravitation acceleration, body temperature, heart rate variability) | Combined (photo, mechanical, electric) | Ear canal | Tertiary |
| Correia et al [69] | 2020 | Combined (cardiovascular, metabolic and mental disorders) | PPG | Photo | Earlobe | Secondary |
| Davies et al [62] | 2020 | Respiration (COVID-19, sleep apnea, hypoxemia) | Others (SpO2) | Photo | Photo | Secondary |
| Hossain et al [38] | 2020 | Diet related (mastication) | Combined (EMG, ear canal pressure and strain, muscle movement) | Combined (air pressure, piezoelectric strain, electric) | Ear canal | Primary |
| Jorgensen et al [53] | 2020 | CNS disease (seizure, epilepsy) | EEG | Electric | Multiple (ear canal, concha) | Secondary |
| Luo et al [72] | 2020 | Others (ovulation, fertility) | Body temperature | Thermal | Ear canal | Primary |
| Nakamura et al [45] | 2020 | Brain related (sleep quality) | EEG | Electric | Ear canal | Secondary |
| Roossien et al [73] | 2020 | Others (heat stress) | Body temperature | Thermal | Inner ear | Secondary |
| Vandecasteele et al [54] | 2020 | CNS disease (seizure, epilepsy) | EEG | Electric | Behind the ear | Secondary |
