# Supplementary material for: Health-Related Indicators Measured Using Earable Devices: Systematic Review
Source: JMIR Mhealth Uhealth. 2022 Nov 15;10(11):e36696. doi: 10.2196/36696 (PMC9709679; doi:10.2196/36696)
Supplement: Multimedia Appendix 4 [file mhealth_v10i11e36696_app4.docx]

**Multimedia Appendix 4. Key properties of earable devices and health-related indicators.**

Supplementary Figure 1. Annual trend of earable device research


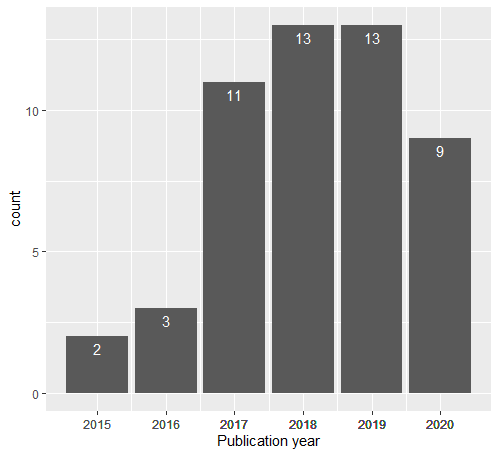


Only few articles were found in 2015 and 2016. The number of articles sharply increased since 2017 and remained similar.

Supplementary Figure 2. Annual trend of health-related indicators in earable device research


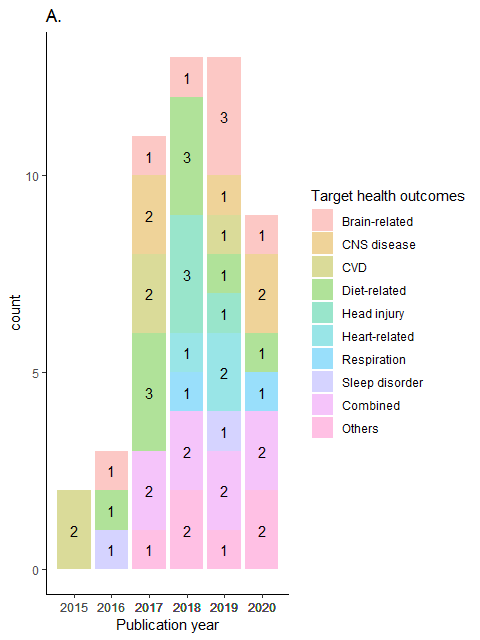

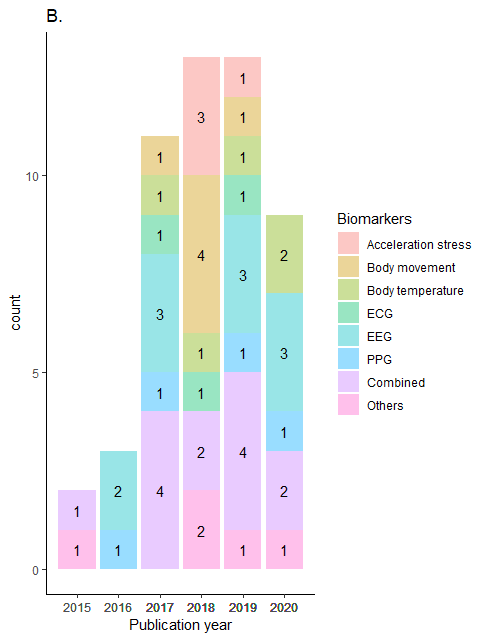

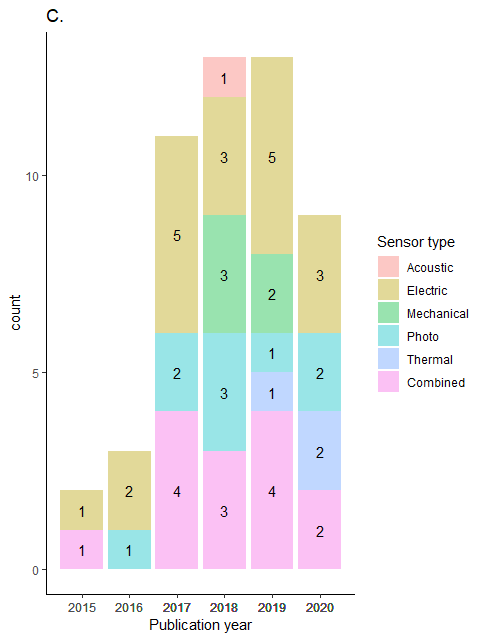

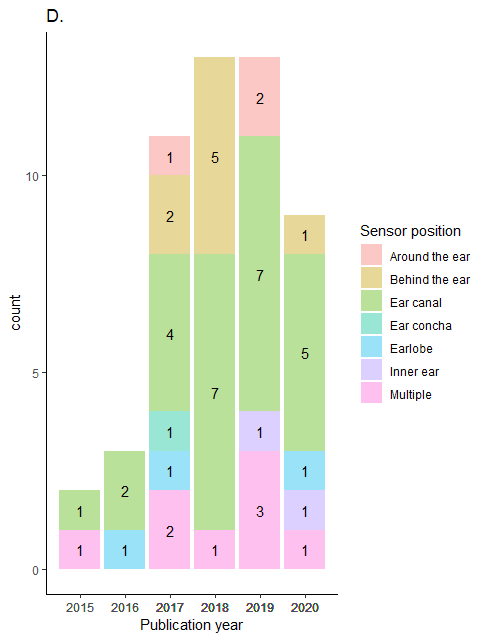

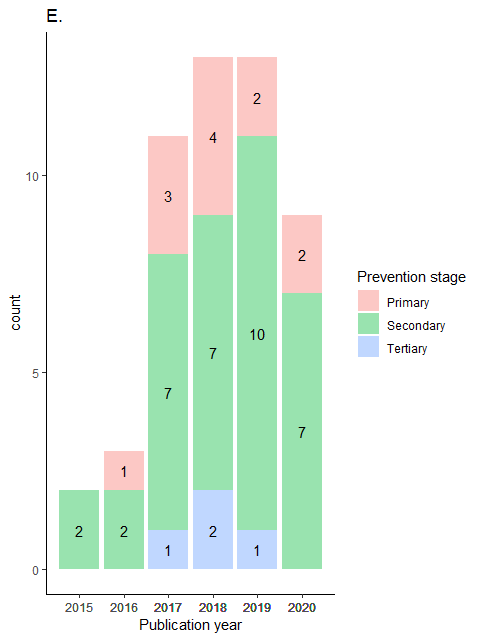


Research topics diversified mainly with regard to (A) target health outcomes and (B) biomarkers, followed by (C) sensor types. (D) Position of the device remained mostly in-ear form. (E) Prevention stage was dominantly secondary.
